# Supplementary material for: DNA Structure Design Is Improved Using an Artificially Expanded Alphabet of Base Pairs Including Loop and Mismatch Thermodynamic Parameters
Source: ACS Synth Biol. 2023 Sep 6;12(9):2750–63. doi: 10.1021/acssynbio.3c00358 (PMC10510751; doi:10.1021/acssynbio.3c00358)
Supplement: Supplementary file 1 — sb3c00358_si_001.pdf [file sb3c00358_si_001.pdf]

Supplementary Materials to Accompany:

DNA Structure Design Is Improved Using an Artificially Expanded Alphabet of Base Pairs Including Loop and Mismatch Thermodynamic Parameters

Tuan M. Pham[1,§], Terrel Miffin[2,§], Hongying Sun[3,§], Kenneth K. Sharp[2], Xiaoyu Wang[2], Mingyi Zhu[1], Shuichi Hoshika[4], Raymond J. Peterson[5], Steven A. Benner[4], Jason D. Kahn[2*], and David H. Mathews[1*]

1. Department of Biochemistry & Biophysics and Center for RNA Biology, University of Rochester Medical Center, Rochester, NY
2. Department of Chemistry & Biochemistry, University of Maryland, College Park, MD
3. Department of Surgery, University of Rochester Medical Center, Rochester, NY
4. Foundation for Applied Molecular Evolution, Alachua, FL
5. DNA Analytics, Greenbelt, MD
* Correspondence to jdkahn@umd.edu or David_Mathews@urmc.rochester.edu

§ T.M.P., T.M., and H.S. contributed equally to this paper.

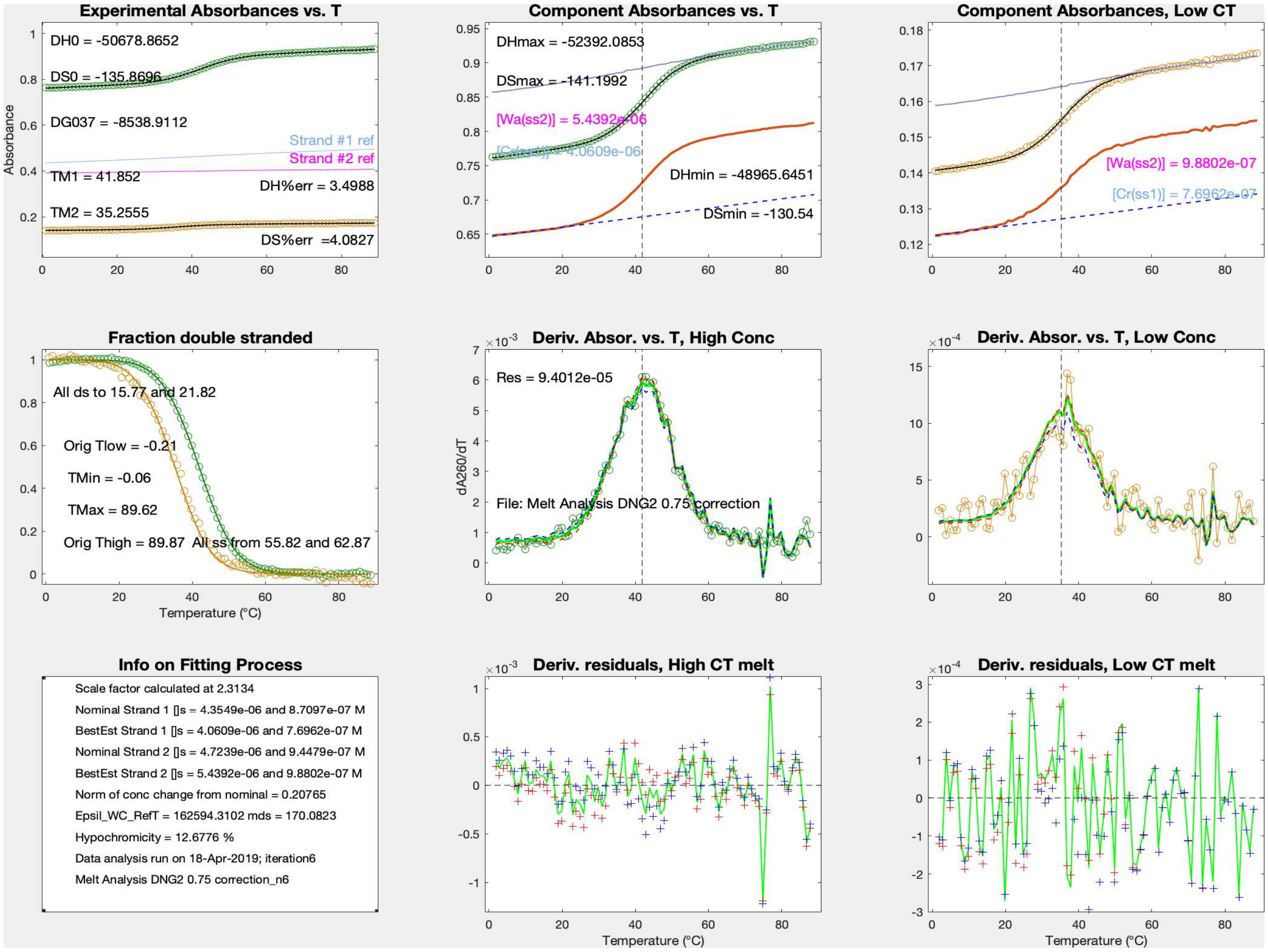

Figure S1. Sample output from global fitting to two optical melting experiments, shown as a screen shot of the output from the Matlab routines. The plots show the raw data, derivative plots, and analysis for two concentrations. Wa (short for Watson) and Cr (short for Crick) refer to the two strands. The Wa strand is in excess, by definition. Nominal concentrations of the working stocks are determined from strand #1 and strand #2 reference curves in the top left plot. The lower orange curves in the two plots at the top right show the experimental curve minus the absorbance due to the excess single strand. Note that the *y*-axis does not start at zero. Also, note that because of LeChatelier's principle, the orange curve would not actually be observed for equal single strand concentrations. In the algorithm, the global fit to normalized absorbance change weights the higher-concentration curve more heavily, according to the square root of total absorbance. The Matlab routines generate four analogous plots for the four possible combinations of which strand is in excess in each melt. Usually the best-fitting plot is obvious based on the minimum concentration error and the minimum residual error between the fits and the data.

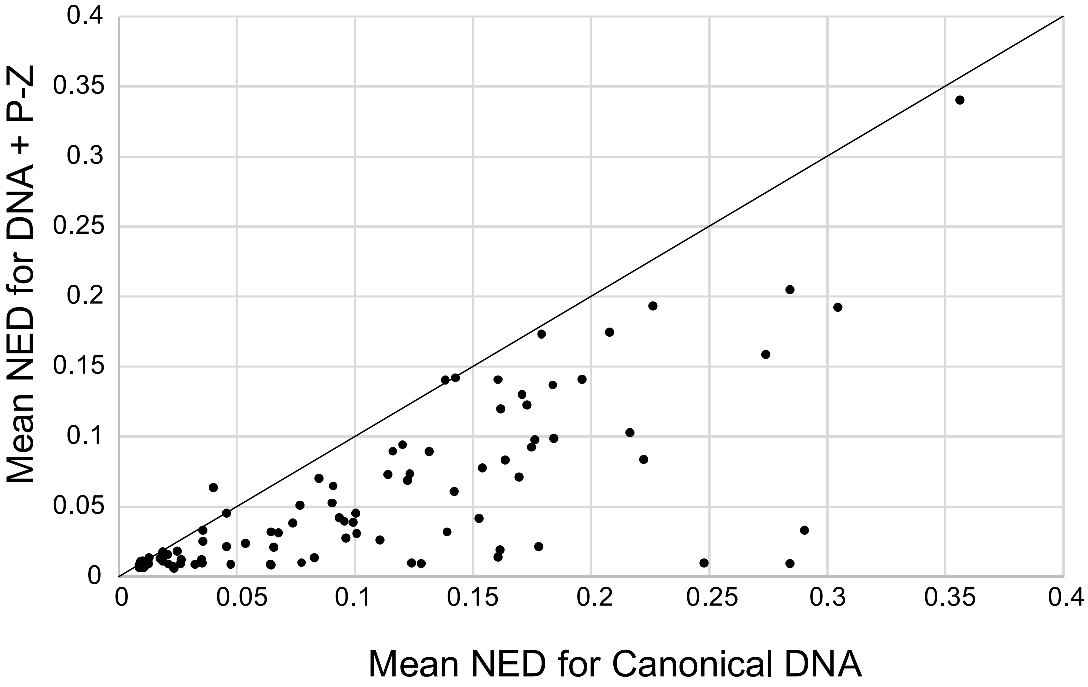

Figure S2. Mean NED is significantly improved with the incorporation of P-Z in designs ($P=1.0\times10^{-13}$). The mean NED for designed sequences that include P-Z pairs as a function of NED for designed sequences that use only canonical DNA nucleotides. Points below the diagonal (shown for reference) are test examples with improved (lower) NED when P-Z pairs are used.

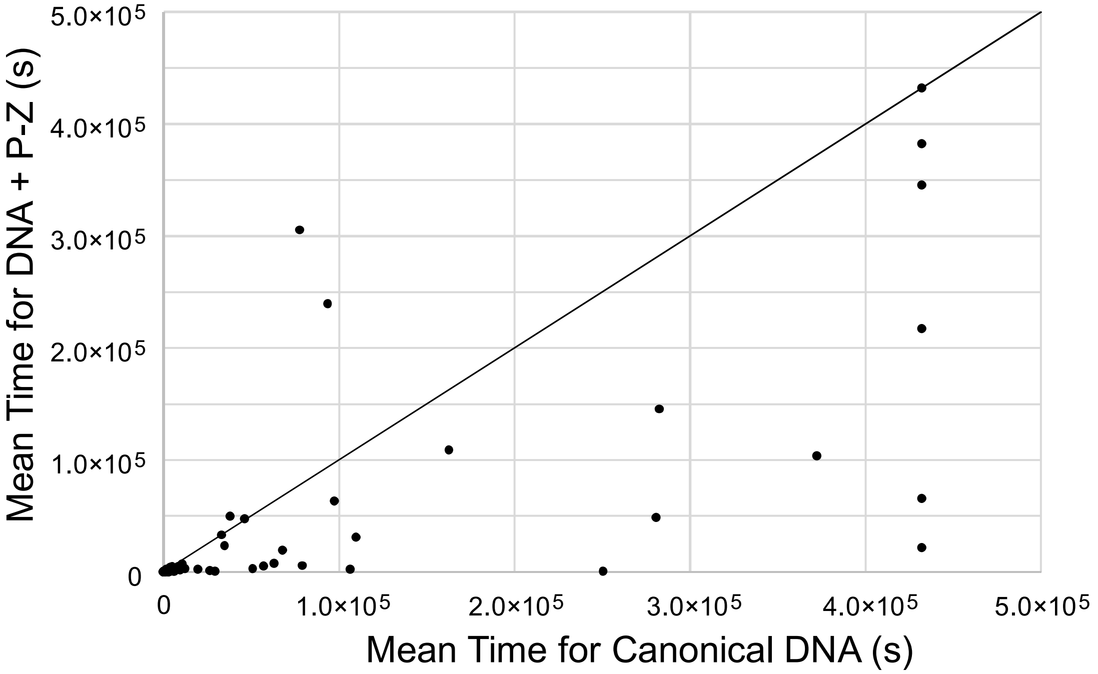

Figure S3. Average time is significantly improved with the use of P-Z pairs as compared to using only canonical DNA nucleotides ($P=1.7\times10^{-3}$). The mean time for five Design calculations with P-Z pairs is plotted as a function of mean time for five Design calculations using only canonical DNA nucleotides. Each point is a single problem from the Eterna 100 set. Failed Designs are those that reached the 5 day limit (432,000 seconds). Six problems were not solved in the five attempts using DNA only, and these are points that align vertically at 432,000 s. One problem was not solved using either DNA or with DNA including PZ, and this point is on the diagonal at 432,000 s. 70 problems were quick (less than 3 hours or 10,800 seconds) to solve for both P-Z and canonical DNA only. These points are clustered in the lower left of the plot.

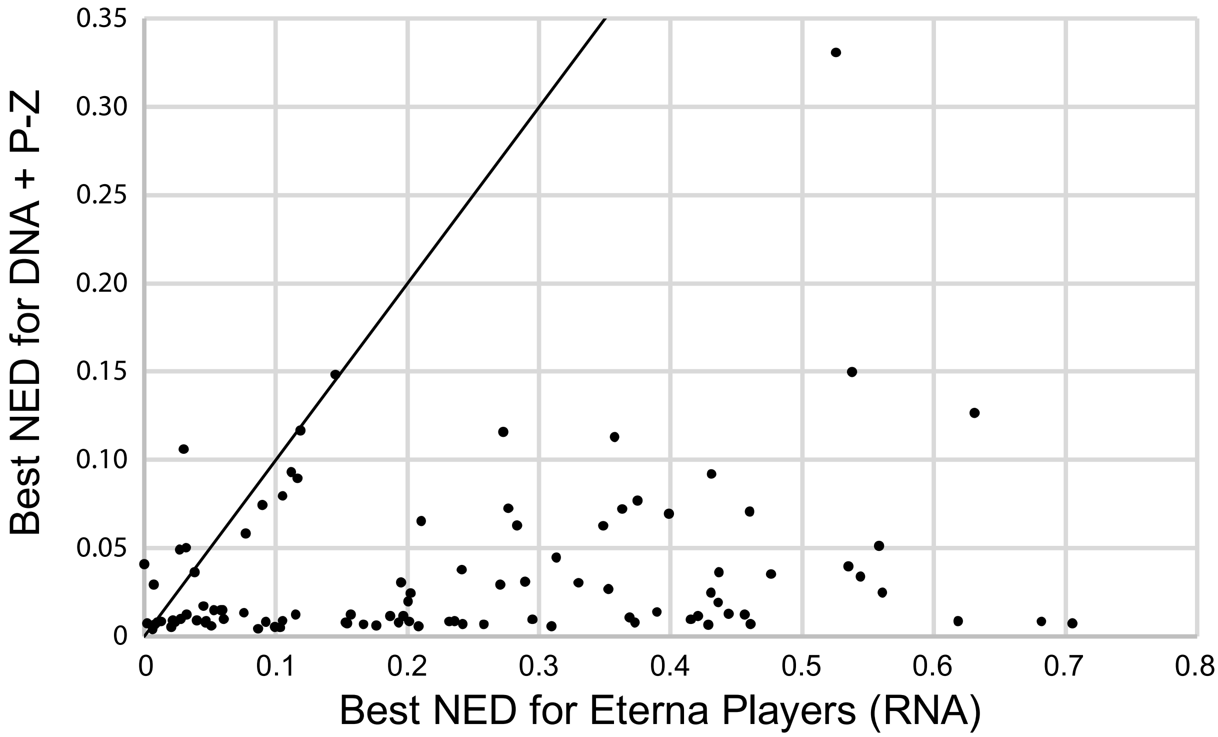

Figure S4. The best NED designed for DNA with P-Z pairs is significantly better than the best NED designed for RNA by Eterna Players (4.28×10^{-19}). The diagonal is shown as a visual aid. Solutions below the diagonal have lower (better) NED for RNAstructure Design than for Eterna players.

Table S1. Optical melting data for 13 new duplexes designed to determine P-Z stack nearest neighbor parameters.

Sequence 1 5′->3′	Sequence 2 3′->5′	ΔH° (kcal/mol)	ΔS° (cal mol^{-1} K^{-1})	$\Delta G°_{37}$ (kcal/mol)
GCACAGATC**P**	CGTGTCTAG**Z**	-75.4 ± 2.9	-205.3 ± 9.0	-11.71 ± 0.15
GCACAGTTT**Z**	CGTGTCAAA**P**	-72.4 ± 2.4	-199.4 ± 7.5	-10.61 ± 0.10
GCACA**P**CTGA	CGTGT**Z**GACT	-72.5 ± 3.3	-194.2 ± 9.9	-12.25 ± 0.20
GCACA**PP**TGA	CGTGT**ZZ**ACT	-76.5 ± 3.6	-203.4 ± 10.7	-13.44 ± 0.25
GCACA**ZP**GGA	CGTGT**PZ**CCT	-80.3 ± 3.5	-212.5 ± 10.3	-14.41 ± 0.27
GCACC**PZ**TGA	CGTGG**ZP**ACT	-74.6 ± 2.8	-195.1 ± 8.3	-14.10 ± 0.21
GCACG**PZ**GAA	CGTGC**ZP**CTT	-75.8 ± 4.5	-198.8 ± 13.3	-14.12 ± 0.35
GCACT**P**ATGA	CGTGA**Z**TACT	-70.3 ± 3.2	-191.1 ± 9.9	-11.03 ± 0.14
GCACT**P**TTGA	CGTGA**Z**AACT	-66.2 ± 2.6	-178.0 ± 7.9	-11.04 ± 0.13
GCACT**ZP**GAA	CGTGA**PZ**CTT	-78.4 ± 4.5	-208.7 ± 13.6	-13.67 ± 0.33
GCACT**ZZ**GAA	CGTGA**PP**CTT	-67.9 ± 3.7	-178.2 ± 11.2	-12.67 ± 0.25
**P**CACAGATGA	**Z**GTGTCTACT	-70.0 ± 2.3	-190.4 ± 7.0	-10.94 ± 0.10
**Z**CACAGATGA	**P**GTGTCTACT	-66.2 ± 2.3	-180.8 ± 7.3	-10.18 ± 0.08

Table S2. The nearest neighbor stack parameters for P-Z pairs with and without $(ZGCATGCP)_2$, which we identified as an outlier. These fits include the terminal P-Z penalty, which we determined to not be required.

Parameter:	Including $(ZGCATGCP)_2$ $\Delta G°_{37}$ (kcal/mol)	Excluding $(ZGCATGCP)_2$ $\Delta G°_{37}$ (kcal/mol)
TP<br>AZ	-1.29 ± 0.17	-1.45 ± 0.15
AP<br>TZ	-1.60 ± 0.19	-1.85 ± 0.16
AZ<br>TP	-1.81 ± 0.21	-1.45 ± 0.19
TZ<br>AP	-1.83 ± 0.18	-1.50 ± 0.17
GZ<br>CP	-2.06 ± 0.20	-1.75 ± 0.18
GP<br>CZ	-2.03 ± 0.19	-2.27 ± 0.16
PZ<br>ZP	-2.12 ± 0.37	-1.58 ± 0.33
CZ<br>GP	-2.33 ± 0.16	-2.08 ± 0.14
PP<br>ZZ	-2.45 ± 0.17	-2.36 ± 0.14
CP<br>GZ	-2.35 ± 0.17	-2.76 ± 0.17
ZP<br>PZ	-2.70 ± 0.39	-3.33 ± 0.35
P end penalty<br>Z	0.33 ± 0.21	0.03 ± 0.19

Table S3. The full set of duplexes used to fit P-Z pairs and the residuals of the fits.

Sequence 1 5′ -> 3′	Sequence 2 3′ -> 5′	Source†	Experimental $\Delta G°_{37}$ (kcal/mol)[A]	Experimental $\Delta G°_{37}$ for P-Z stacks (kcal/mol)[B]	Fit $\Delta G°_{37}$ for P-Z stacks (kcal/mol)[C]	Residual as (Experimental $\Delta G°_{37}$ for P-Z stacks) – (Fit $\Delta G°_{37}$) (kcal/mol)
$CPGATCZG_2$		A	-10.52	-9.45	-9.68	0.23
$CPPATZZG_2$		A	-12.07	-13.60	-13.20	-0.40
$CZACGTPG_2$		A	-10.23	-7.66	-7.05	-0.61
$CZCATGPG_2$		A	-9.87	-8.40	-8.71	0.31
$CZTCGAPG_2$		A	-10.4	-8.03	-7.85	-0.18
$GACPZGTC_2$		A	-10.38	-7.41	-7.10	-0.31
$GACZPGTC_2$		A	-10.79	-7.82	-7.50	-0.32
$GAPATZTC_2$		A	-7.96	-6.89	-6.67	-0.22
$GAZATPTC_2$		A	-7.13	-6.06	-5.77	-0.29
$GAZCGPTC_2$		A	-9.92	-7.55	-7.44	-0.11
$GAZTAPTC_2$		A	-7.23	-6.46	-6.57	0.11
$GAZZPPTC_2$		A	-11.87	-11.70	-10.93	-0.77
$GCAPZTGC_2$		A	-10.48	-5.51	-5.27	-0.24
$GCAZPTGC_2$		A	-10.99	-6.02	-6.23	0.21
$GCPATZGC_2$		A	-11.46	-8.59	-8.50	-0.09
$GCPTAZGC_2$		A	-10.57	-8.00	-8.40	0.40
$GCTPZAGC_2$		A	-8.83	-4.26	-4.47	0.21
$GCTZPAGC_2$		A	-9.73	-5.16	-6.32	1.16
$GGAPZTCC_2$		B	-9.65	-5.88	-5.27	-0.61
$GGAPZTCC_2$		A	-8.63	-4.86	-5.27	0.41
$GGAZPTCC_2$		B	-10.1	-6.33	-6.23	-0.10
$GGAZPTCC_2$		A	-9.66	-5.89	-6.23	0.34
$GGZATPCC_2$		A	-7.93	-5.86	-6.37	0.51
$GGZTAPCC_2$		A	-8.38	-6.61	-7.17	0.56
$GPACGTZC_2$		B	-10.5	-7.93	-7.54	-0.39
$GPACGTZC_2$		A	-9.51	-6.94	-7.54	0.60
$GPCATGZC_2$		A	-8.59	-7.12	-8.04	0.92
$GTGPZCAC_2$		A	-10.28	-6.91	-6.14	-0.77
$GTGZPCAC_2$		A	-10.81	-7.44	-6.83	-0.61
$GTPPZZAC_2$		A	-8.13	-7.76	-9.17	1.41
$GTZATPAC_2$		A	-7.78	-6.51	-5.87	-0.64
$GTZCGPAC_2$		A	-10.65	-8.08	-7.54	-0.54
$GZGATCPC_2$		A	-10.39	-9.32	-9.00	-0.32
$GZZATPPC_2$		A	-9.86	-11.39	-11.07	-0.32
$GZZTAPPC_2$		A	-9.9	-11.73	-11.87	0.14
$PACTAGTZ_2$		A	-6.4	-2.83	-2.97	0.14

$PGACGTCZ_2$		B	-10.2	-5.03	-4.15	-0.88
$PGACGTCZ_2$		A	-9.05	-3.88	-4.15	0.27
$PGCATGCZ_2$		A	-8.61	-2.74	-4.15	1.41
$ZACTAGTP_2$		A	-6.33	-2.76	-2.90	0.14
GCACAGATCP	CGTGTCTAGZ	C	-11.71	-2.31	-2.77	0.45
GCACAGTTTZ	CGTGTCAAAP	C	-10.61	-1.31	-1.49	0.17
GCACAPCTGA	CGTGTZGACT	C	-12.25	-3.55	-3.59	0.03
GCACAPPTGA	CGTGTZZACT	C	-13.44	-6.04	-5.64	-0.40
GCACAZPGGA	CGTGTPZCCT	C	-14.4	-6.70	-6.86	0.16
GCACCPZTGA	CGTGGZPACT	C	-14.1	-6.40	-6.19	-0.21
GCACGPZGAA	CGTGCZPCTT	C	-14.12	-6.52	-6.62	0.10
GCACTPATGA	CGTGAZTACT	C	-11.02	-2.92	-2.93	0.01
GCACTPTTGA	CGTGAZAACT	C	-11.04	-2.84	-2.89	0.05
GCACTZPGAA	CGTGAPZCTT	C	-13.67	-6.97	-6.91	-0.05
GCACTZZGAA	CGTGAPPCTT	C	-12.67	-5.97	-6.60	0.63
GCCAPTTAA	CGGTZAATT	B	-9.14	-3.04	-3.29	0.25
GCCAPTTAA	CGGTZAATT	A	-9.01	-2.91	-3.29	0.38
GCZAGTTAA	CGPTCAATT	B	-9.38	-3.88	-3.52	-0.36
GCZAGTTAA	CGPTCAATT	A	-9.19	-3.69	-3.52	-0.17
GZCAGTTAA	CPGTCAATT	A	-9.35	-4.55	-4.02	-0.53
GZCAGTTAA	CPGTCAATT	B	-9.23	-4.43	-4.02	-0.41
GZZAGTTAA	CPPTCAATT	B	-9.59	-6.29	-5.54	-0.76
GZZAGTTAA	CPPTCAATT	A	-8.45	-5.15	-5.54	0.39
PCACAGATGA	ZGTGTCTACT	C	-10.94	-2.24	-1.74	-0.50
ZCACAGATGA	PGTGTCTACT	C	-10.18	-1.48	-2.28	0.80

[†]Sources of data are A, Hoshika et al.[15]; B, Wang et al.[16]; C, this work.

[A] Experimental $\Delta G°_{37}$ (kcal/mol) is the free energy change at 37 °C for the duplex as measured by optical melting.

[B] Experimental $\Delta G°_{37}$ for P-Z stacks (kcal/mol) is the free energy change at 37 °C that is attributed to stacks with P-Z pairs, i.e. it is the Experimental $\Delta G°_{37}$ with canonical DNA stacks and the intermolecular initiation subtracted. This is the target value for the fits because only stacks with P-Z pairs are fit.

[C] Fit $\Delta G°_{37}$ for P-Z stacks (kcal/mol) is the free energy change at 37 °C estimated by the fit P-Z stack parameters. Deviations between this and Experimental $\Delta G°_{37}$ for P-Z stacks (kcal/mol) constitute the residuals of the fit.

Table S4. Optical melting data for duplexes designed to determine G-Z stack nearest neighbor parameters. Eleven are new sequences for this work. Uncertainties are the fit uncertainties from simultaneous fitting to melting experiments done at two concentrations as described in the Materials and Methods.

Sequence 1 5′->3′	Sequence 2 3′->5′	$\Delta H°$ (kcal/mol)	$\Delta S°$ (cal mol^{-1} K^{-1})	$\Delta G°_{37}$ (kcal/mol)	Source†/ comment
AGCCAGTTAZ	TCGGTCAATG	-63.6 ± 1.2	-171.9 ± 3.7	-10.27 ± 0.09	This work
GCCACGZAAG	CGGTGZGTTC	-48.5 ± 2.0	-128.3 ± 6.3	-8.69 ± 0.06	This work
GCCAGAZAAG	CGGTZTGTTC	-52.1 ± 1.9	-140.0 ± 5.8	-8.68 ± 0.06	This work; duplicate runs
GCCAGAZAAG	CGGTZTGTTC	-50.7 ± 1.8	-135.9 ± 5.5	-8.54 ± 0.05	
GCCAGCGPAG	CGGTCGZZTC	-63.9 ± 7.2	-167.1 ± 21.8	-12.09 ± 0.44	This work
GCCAGTTAA	CGGTZAATT	-47.9 ± 2.4	-129.2 ± 7.8	-7.83 ± 0.07	Wang, 5ZG
GCCAGZZPAG	CGGTCGGZTC	-63.6 ± 8.7	-168.8 ± 26.6	-11.25 ± 0.43	This work
GCCAZZGAAG	CGGTGGZTTC	-41.9 ± 2.1	-109.1 ± 6.5	-8.05 ± 0.06	This work
GCCGZTTAAG	CGGZPAATTC	-72.5 ± 1.8	-197.2 ± 5.6	-11.36 ± 0.10	This work
GCZAGTTAA	CGGTCAATT	-49.6 ± 3.4	-137.0 ± 10.9	-7.07 ± 0.02	Wang, 3ZG
GCZCGTTAAG	CGGGZAATTC	-50.0 ± 1.5	-135.4 ± 4.8	-7.98 ± 0.06	This work
GGCGGTTAAG	CZGZCAATTC	-59.1 ± 4.5	-164.5 ± 14.4	-8.08 ± 0.02	This work
GZCAGTTAA	CGGTCAATT	-52.8 ± 3.6	-146.0 ± 11.9	-7.52 ± 0.14	Wang, 2ZG
GZPAGTTAAG	CGZTCAATTC	-63.8 ± 1.4	-173.3 ± 4.4	-10.01 ± 0.07	This work
GZZAGTTAA	CGGTCAATT	-46.3 ± 2.8	-128.4 ± 9.3	-6.48 ± 0.07	Wang, 2,3ZG
GZZZGTTAAG	CGGPCAATTC	-63.4 ± 3.5	-171.0 ± 10.7	-10.35 ± 0.15	This work

†Wang et al., 2017;[16] uncertainty estimates for $\Delta G°_{37}$ from Wang, 2016.[55]

Table S5. The set of duplexes used to fit G-Z pair stacking nearest neighbor parameters and the residuals of the fits. Note that the residuals are 0 kcal/mol in most cases because there was no redundancy in the set of dinucleotides with G-Z pair stacks.

Sequence 1 5′ -> 3′	Sequence 2 3′ -> 5′	Experimental ΔG°_{37} (kcal/mol)	Experimental ΔG°_{37} for G-Z stacks (kcal/mol)	Fit ΔG°_{37} for G-Z stacks (kcal/mol)	Residual as (Experimental ΔG°_{37}) – (Fit ΔG°_{37}) (kcal/mol)
AGCCAGTTAZ	TCGGTCAATG	-10.27	-1.17	-1.17	0.00
GCCACGZAAG	CGGTGZGTTC	-8.69	-1.49	-1.49	0.00
GCCAGAZAAG	CGGTZTGTTC	-8.68	-2.88	-2.81	-0.07
GCCAGAZAAG	CGGTZTGTTC	-8.54	-2.74	-2.81	0.07
GCCAGCGPAG	CGGTCGZZTC	-12.09	-2.30	-2.30	0.00
GCCAGTTAA	CGGTZAATT	-7.84	-1.74	-1.74	0.00
GCCAGZZPAG	CGGTCGGZTC	-11.25	-3.66	-3.66	0.00
GCCAZZGAAG	CGGTGGZTTC	-8.05	-2.25	-2.25	0.00
GCCGZTTAAG	CGGZPAATTC	-11.36	-3.61	-3.61	0.00
GCZAGTTAA	CGGTCAATT	-7.07	-1.57	-1.57	0.00
GCZCGTTAAG	CGGGZAATTC	-7.98	-3.88	-3.88	0.00
GGCGGTTAAG	CZGZCAATTC	-8.08	-4.78	-4.78	0.00
GZCAGTTAA	CGGTCAATT	-7.61	-2.81	-2.81	0.00
GZPAGTTAAG	CGZTCAATTC	-10.01	-3.92	-3.92	0.00
GZZAGTTAA	CGGTCAATT	-6.50	-3.20	-3.20	0.00
GZZZGTTAAG	CGGPCAATTC	-10.35	-4.28	-4.28	0.00

Table S6. Stacking enthalpy and entropy change parameters for stacks with P-Z pairs.

Parameter	ΔH° (kcal/mol)	ΔS° (e.u.)
TP<br>AZ	-6.33 ± 1.45	-14.83 ± 4.34
AP<br>TZ	-8.22 ± 1.60	-19.74 ± 4.77
AZ<br>TP	-4.96 ± 1.63	-11.04 ± 4.88
TZ<br>AP	-7.36 ± 1.45	-19.13 ± 4.34
GZ<br>CP	-2.23 ± 1.61	-0.98 ± 4.80
PZ<br>ZP	-3.63 ± 3.23	-6.67 ± 9.64
GP<br>CZ	-9.23 ± 1.57	-21.51 ± 4.69
CZ<br>GP	-6.80 ± 1.27	-14.92 ± 3.79
PP<br>ZZ	-10.96 ± 1.32	-27.45 ± 3.94
CP<br>GZ	-12.32 ± 1.61	-30.24 ± 4.80
ZP<br>PZ	-15.43 ± 3.18	-37.92 ± 9.51

Table S7A. The full set of duplexes used to fit P-Z stacking enthalpy change parameters and the residuals of the fits.

Sequence 1 5′ -> 3′	Sequence 2 3′ -> 5′	Source†	Experimental $\Delta H°_{37}$ (kcal/mol)	Experimental $\Delta H°_{37}$ for P-Z stacks (kcal/mol)	Fit $\Delta H°_{37}$ for P-Z stacks (kcal/mol)	Residual as (Experimental $\Delta H°_{37}$) – (Fit $\Delta H°_{37}$) (kcal/mol)
$CPGATCZG_2$		A	-62.9	-39.5	-38.2	-1.2
$CPPATZZG_2$		A	-71.2	-64.2	-61.3	-2.9
$CZACGTPG_2$		A	-57.8	-30.6	-26.3	-4.3
$CZCATGPG_2$		A	-51.1	-27.0	-32.1	5.0
$CZTCGAPG_2$		A	-61.1	-34.3	-30.0	-4.2
$GACPZGTC_2$		A	-57.7	-24.7	-28.3	3.6
$GACZPGTC_2$		A	-57.0	-24.0	-29.0	5.1
$GAPATZTC_2$		A	-59.9	-36.5	-31.1	-5.3
$GAZATPTC_2$		A	-45.7	-22.3	-22.6	0.3
$GAZCGPTC_2$		A	-52.4	-25.6	-28.4	2.8
$GAZTAPTC_2$		A	-46.0	-22.6	-26.4	3.8
$GAZZPPTC_2$		A	-66.0	-49.8	-47.3	-2.5
GCACAGATCP	CGTGTCTAGZ	C	-75.4	-9.0	-12.3	3.3
GCACAGTTTZ	CGTGTCAAAP	C	-72.4	-6.0	-7.4	1.3
GCACAPCTGA	CGTGTZGACT	C	-72.5	-15.2	-10.4	-4.7
GCACAPPTGA	CGTGTZZACT	C	-76.5	-27.0	-24.1	-2.9
GCACAZPGGA	CGTGTPZCCT	C	-80.3	-31.3	-27.2	-4.1
GCACCPZTGA	CGTGGZPACT	C	-74.6	-25.6	-24.2	-1.4
GCACGPZGAA	CGTGCZPCTT	C	-75.8	-25.1	-25.2	0.1
GCACTPATGA	CGTGAZTACT	C	-70.3	-14.3	-13.7	-0.6
GCACTPTTGA	CGTGAZAACT	C	-66.2	-9.8	-11.3	1.4
GCACTZPGAA	CGTGAPZCTT	C	-78.4	-30.5	-29.6	-0.9
GCACTZZGAA	CGTGAPPCTT	C	-67.9	-20.0	-30.6	10.6
$GCAPZTGC_2$		A	-60.2	-23.7	-20.1	-3.7
$GCAZPTGC_2$		A	-64.6	-28.2	-25.4	-2.8
GCCAPTTAA	CGGTZAATT	B	-56.2	-9.9	-13.2	3.3
GCCAPTTAA	CGGTZAATT	A	-58.5	-12.2	-13.2	1.0
$GCPATZGC_2$		A	-67.1	-40.5	-39.4	-1.1
$GCPTAZGC_2$		A	-61.9	-35.3	-34.6	-0.7
$GCTPZAGC_2$		A	-46.0	-11.0	-16.3	5.3
$GCTZPAGC_2$		A	-55.7	-20.7	-30.1	9.5
GCZAGTTAA	CGPTCAATT	B	-58.5	-12.5	-13.1	0.7
GCZAGTTAA	CGPTCAATT	A	-65.1	-19.1	-13.1	-6.0

$GGAPZTCC_2$		A	-45.3	-13.1	-20.1	7.0
$GGAPZTCC_2$		B	-58.1	-25.9	-20.1	-5.8
$GGAZPTCC_2$		A	-55.0	-22.8	-25.4	2.6
$GGAZPTCC_2$		B	-60.4	-28.2	-25.4	-2.8
$GGZATPCC_2$		A	-36.5	-13.5	-17.1	3.7
$GGZTAPCC_2$		A	-36.4	-13.4	-20.9	7.5
$GPACGTZC_2$		A	-57.3	-30.1	-33.2	3.1
$GPACGTZC_2$		B	-67.3	-40.1	-33.2	-6.9
$GPCATGZC_2$		A	-39.7	-15.7	-22.9	7.3
$GTGPZCAC_2$		A	-59.2	-25.6	-22.1	-3.5
$GTGZPCAC_2$		A	-57.5	-23.9	-19.9	-4.0
$GTPPZZAC_2$		A	-56.4	-39.8	-38.2	-1.5
$GTZATPAC_2$		A	-52.7	-28.9	-27.4	-1.5
$GTZCGPAC_2$		A	-62.3	-35.1	-33.2	-1.9
GZCAGTTAA	CPGTCAATT	B	-58.5	-13.8	-11.5	-2.3
GZCAGTTAA	CPGTCAATT	A	-71.6	-26.9	-11.5	-15.4
$GZGATCPC_2$		A	-56.4	-33.0	-29.1	-3.9
GZZAGTTAA	CPPTCAATT	B	-58.3	-22.1	-19.5	-2.5
GZZAGTTAA	CPPTCAATT	A	-58.7	-22.5	-19.5	-2.9
$GZZATPPC_2$		A	-43.4	-36.4	-39.0	2.6
$GZZTAPPC_2$		A	-46.7	-39.7	-42.8	3.2
$PACTAGTZ_2$		A	-52.2	-12.8	-14.7	1.9
PCACAGATGA	ZGTGTCTACT	C	-70.0	-7.1	-2.2	-4.9
$PGACGTCZ_2$		A	-54.7	-11.1	-13.6	2.5
$PGACGTCZ_2$		B	-69.3	-25.7	-13.6	-12.1
$PGCATGCZ_2$		A	-42.8	0.8	-13.6	14.4
$ZACTAGTP_2$		A	-51.7	-12.3	-12.7	0.4
ZCACAGATGA	PGTGTCTACT	C	-66.2	-3.3	-9.2	5.9

†Sources of data are A, Hoshika et al.[15]; B, Wang et al.[16]; C, this work.

Table S7B. The full set of duplexes used to fit P-Z stacking entropy change parameters and the residuals of the fits.

Sequence 1 5′ -> 3′	Sequence 2 3′ -> 5′	Source†	Experimental $\Delta S°_{37}$ (e.u.)	Experimental $\Delta S°_{37}$ for P-Z stacks (e.u.)	Fit $\Delta S°_{37}$ for P-Z stacks (e.u.)	Residual as (Experimental $\Delta S°_{37}$) – (Fit $\Delta S°_{37}$) (e.u)
$CPGATCZG_2$		A	-168.7	-96.8	-90.3	-6.5
$CPPATZZG_2$		A	-190.7	-163.2	-153.6	-9.5
$CZACGTPG_2$		A	-153.2	-74.1	-59.5	-14.6
$CZCATGPG_2$		A	-132.8	-59.9	-72.9	13.0
$CZTCGAPG_2$		A	-163.3	-84.6	-69.3	-15.3
$GACPZGTC_2$		A	-152.6	-56.3	-67.1	10.9
$GACZPGTC_2$		A	-148.8	-52.5	-67.8	15.2
$GAPATZTC_2$		A	-167.3	-95.4	-77.8	-17.7
$GAZATPTC_2$		A	-124.2	-52.3	-51.7	-0.6
$GAZCGPTC_2$		A	-137.0	-58.3	-65.1	6.8
$GAZTAPTC_2$		A	-125.0	-52.2	-61.6	9.4
$GAZZPPTC_2$		A	-174.4	-122.9	-114.9	-8.0
GCACAGATCP	CGTGTCTAGZ	C	-205.3	-21.6	-30.2	8.6
GCACAGTTTZ	CGTGTCAAAP	C	-199.4	-15.5	-19.1	3.7
GCACAPCTGA	CGTGTZGACT	C	-194.2	-29.0	-20.7	-8.3
GCACAPPTGA	CGTGTZZACT	C	-203.4	-59.2	-58.2	-1.0
GCACAZPGGA	CGTGTPZCCT	C	-212.5	-71.1	-63.9	-7.3
GCACCPZTGA	CGTGGZPACT	C	-195.1	-53.7	-56.7	2.9
GCACGPZGAA	CGTGCZPCTT	C	-198.8	-51.5	-58.4	7.0
GCACTPATGA	CGTGAZTACT	C	-191.1	-28.2	-34.0	5.8
GCACTPTTGA	CGTGAZAACT	C	-178.0	-14.2	-25.9	11.7
GCACTZPGAA	CGTGAPZCTT	C	-208.7	-67.6	-72.0	4.3
GCACTZZGAA	CGTGAPPCTT	C	-178.2	-37.1	-76.8	39.7
$GCAPZTGC_2$		A	-160.2	-58.9	-46.2	-12.7
$GCAZPTGC_2$		A	-172.7	-71.4	-60.0	-11.4
GCCAPTTAA	CGGTZAATT	B	-151.7	-13.7	-30.8	17.1
GCCAPTTAA	CGGTZAATT	A	-159.6	-21.6	-30.8	9.2
$GCPATZGC_2$		A	-179.4	-103.1	-98.7	-4.4
$GCPTAZGC_2$		A	-165.3	-88.1	-82.6	-5.6
$GCTPZAGC_2$		A	-119.9	-22.0	-36.3	14.4
$GCTZPAGC_2$		A	-148.1	-50.2	-76.2	26.0
GCZAGTTAA	CGPTCAATT	B	-158.2	-19.4	-29.8	10.3
GCZAGTTAA	CGPTCAATT	A	-180.3	-41.5	-29.8	-11.7

GGAPZTCC$_2$		A	-118.2	-26.9	-46.2	19.2
GGAPZTCC$_2$		B	-156.2	-64.9	-46.2	-18.8
GGAZPTCC$_2$		A	-146.0	-54.7	-60.0	5.3
GGAZPTCC$_2$		B	-162.2	-70.9	-60.0	-10.9
GGZATPCC$_2$		A	-92.0	-24.7	-31.6	7.0
GGZTAPCC$_2$		A	-90.2	-22.0	-41.5	19.5
GPACGTZC$_2$		A	-154.1	-745.0	-81.3	6.3
GPACGTZC$_2$		B	-183.1	-104.0	-81.3	-22.8
GPCATGZC$_2$		A	-100.2	-27.3	-45.0	17.7
GTGPZCAC$_2$		A	-157.6	-60.3	-49.7	-10.6
GTGZPCAC$_2$		A	-150.5	-53.2	-39.9	-13.4
GTPPZZAC$_2$		A	-155.5	-103.6	-91.2	-12.3
GTZATPAC$_2$		A	-144.8	-72.5	-67.9	-4.6
GTZCGPAC$_2$		A	-166.4	-87.3	-81.3	-6.0
GZCAGTTAA	CPGTCAATT	B	-158.9	-21.8	-22.5	0.7
GZCAGTTAA	CPGTCAATT	A	-200.6	-63.5	-22.5	-41.0
GZGATCPC$_2$		A	-148.4	-76.5	-62.4	-14.0
GZZAGTTAA	CPPTCAATT	B	-156.9	-42.5	-43.3	0.8
GZZAGTTAA	CPPTCAATT	A	-161.9	-47.5	-43.3	-4.2
GZZATPPC$_2$		A	-108.1	-80.6	-86.5	5.9
GZZTAPPC$_2$		A	-118.5	-90.1	-96.4	6.3
PACTAGTZ$_2$		A	-147.7	-32.5	-38.3	5.8
PCACAGATGA	ZGTGTCTACT	C	-190.4	-7.0	-1.0	-6.1
PGACGTCZ$_2$		A	-147.2	-23.7	-29.8	6.2
PGACGTCZ$_2$		B	-190.6	-67.1	-29.8	-37.2
PGCATGCZ$_2$		A	-110.1	11.6	-29.8	41.5
ZACTAGTP$_2$		A	-146.3	-31.1	-29.7	-1.4
ZCACAGATGA	PGTGTCTACT	C	-180.8	2.7	-21.5	24.2

†Sources of data are A, Hoshika et al.[15]; B, Wang et al.[16]; C, this work.

Table S8. Optical melting data for eight systems with dangling ends (this work).

Sequence 1<br>5′->3′	Sequence 2<br>3′->5′	ΔH°<br>(kcal/mol)	ΔS°<br>(cal mol^{-1} K^{-1})	$ΔG°_{37}$<br>(kcal/mol)
**P**CACAGATGA	GTGTCTACT	-65.4 ± 2.4	-181.5 ± 7.6	-9.07 ± 0.05
CACAGATGA	**P**GTGTCTACT	-61.4 ± 3.5	-170.2 ± 11.0	-8.63 ± 0.05
**Z**CACAGATGA	GTGTCTACT	-64.3 ± 3.3	-177.2 ± 10.3	-9.34 ± 0.08
CACAGATGA	**Z**GTGTCTACT	-64.3 ± 3.3	-177.2 ± 10.3	-9.34 ± 0.08
GCACAGTTT**Z**	CGTGTCAAA	-60.4 ± 2.8	-164.2 ± 8.8	-9.47 ± 0.08
GCACAGTTT	CGTGTCAAA**Z**	-72.5 ± 2.5	-201.1 ± 7.7	-10.10 ± 0.08
GCACAGTTT**P**	CGTGTCAAA	-61.2 ± 1.2	-167.5 ± 3.7	-9.21 ± 0.03
GCACAGTTT	CGTGTCAAA**P**	-70.8 ± 1.7	-196.3 ± 5.3	-9.93 ± 0.05

Table S9. Optical melting data for thirteen systems with terminal mismatches (this work).

Sequence 1 5′->3′	Sequence 2 3′->5′	ΔH° (kcal/mol)	ΔS° (cal mol^{-1} K^{-1})	ΔG°$_{37}$ (kcal/mol)
**P**CACAGATGA	**P**GTGTCTACT	-61.3 ± 1.6	-168.2 ± 4.9	-9.14 ± 0.03
**Z**CACAGATGA	**Z**GTGTCTACT	-64.1 ± 2.4	-176.2 ± 7.4	-9.46 ± 0.06
GCACAGTTT**Z**	CGTGTCAAA**Z**	-64.5 ± 2.3	-175.2 ± 7.3	-10.21 ± 0.08
GCACAGTTT**P**	CGTGTCAAA**P**	-62.6 ± 2.7	-170.3 ± 8.4	-9.81 ± 0.08
G**Z**CAGTTGAA	G**P**GTCAACTT	-61.9 ± 2.9	-167.9 ± 9.0	-9.81 ± 0.10
G**Z**CAGTTGAA	**P**GTCAACTT	-61.8 ± 3.2	-168.8 ± 9.9	-9.45 ± 0.08
A**Z**CAGTTGAA	A**P**GTCAACTT	-65.0 ± 1.7	-178.6 ± 5.4	-9.56 ± 0.05
A**Z**CAGTTGAA	**P**GTCAACTT	-65.0 ± 1.7	-178.4 ± 5.5	-9.62 ± 0.05
C**Z**CAGTTGAA	C**P**GTCAACTT	-62.7 ± 2.2	-172.3 ± 6.9	-9.28 ± 0.06
C**Z**CAGTTGAA	**P**GTCAACTT	-61.8 ± 2.0	-169.9 ± 6.4	-9.11 ± 0.05
G**Z**CAGTTGAA	A**P**GTCAACTT	-61.7 ± 1.5	-168.3 ± 4.7	-9.52 ± 0.04
T**Z**CAGTTGAA	T**P**GTCAACTT	-61.6 ± 1.3	-168.1 ± 4.1	-9.43 ± 0.03
T**Z**CAGTTGAA	**P**GTCAACTT	-63.5 ± 1.5	-174.7 ± 4.8	-9.36 ± 0.04

Table S10. Optical melting data for systems with single mismatches (from Wang et al., 2017, Table 2)[16] and two tandem mismatches (this work). NA: T_m between 5 °C and 15 °C, so ΔH° and ΔS° are not reliable. Four 9-bp tandem mismatch duplexes from Wang et al. did not show melting transitions with T_m > 5 °C, not shown here.

Sequence 1 5′->3′	Sequence 2 3′->5′	ΔH° (kcal/mol)	ΔS° (cal mol^{-1} K^{-1})	$ΔG°_{37}$ (kcal/mol)	Source†
GCCA**P**TTAA	CGGT**C**AATT	-58.6 ± 2.6	-171.0 ± 8.7	-5.5 ± 0.1	Wang
GC**C**AGTTAA	CG**P**TCAATT	-52.6 ± 2.4	-151.4 ± 8.2	-5.6 ± 0.1	Wang
G**C**CAGTTAA	C**P**GTCAATT	-49.2 ± 2.8	-139.8 ± 9.3	-5.8 ± 0.1	Wang
GCCA**P**TTAA	CGGT**T**AATT	-61.3 ± 3.8	-183.2 ± 12.7	-4.5 ± 0.2	Wang
GC**T**AGTTAA	CG**P**TCAATT	NA	NA	-4.6 ± 0.1	Wang
G**T**CAGTTAA	C**P**GTCAATT	-45.4 ± 3.4	-128.1 ± 11.2	-5.7 ± 0.1	Wang
GCCA**A**TTAA	CGGT**Z**AATT	NA	NA	-4.7 ± 0.2	Wang
GC**Z**AGTTAA	CG**A**TCAATT	NA	NA	NA	Wang
G**Z**CAGTTAA	C**A**GTCAATT	-41.3 ± 3.4	-115.7 ± 11.3	-5.4 ± 0.1	Wang
GCACT**ZZ**GAA	CGTGA**ZZ**CTT	-27.3 ± 8.5	-66.3 ± 27.4	-6.72 ± 0.02	This Work
GCACT**PP**GAA	CGTGA**PP**CTT	-24.4 ± 3.7	-60.1 ± 13.6	-5.72 ± 0.50	This Work

†Source of prior experiments is Wang et al.[16]

Table S11A. 5′ Dangling ends on a Z-P pair as compared to C-G and T-A pairs.

5' dangling end on Z-P (kcal/mol)		5' dangling end on C-G (kcal/mol)		5' dangling end on T-A (kcal/mol)	
5′GZ<br>3′ P	-0.16	5′GC<br>3′ G	-0.7	5′GT<br>3′ A	-0.5
5′AZ<br>3′ P	-0.33	5′AC<br>3′ G	-0.9	5′AT<br>3′ A	-0.5
5′CZ<br>3′ P	0.18	5′CC<br>3′ G	-0.5	5′CT<br>3′ A	-0.2
5′TZ<br>3′ P	-0.07	5′TC<br>3′ G	-0.6	5′TT<br>3′ A	-0.3

Table S11B. P and Z dangling ends compared to canonical nucleotides.

Dangling end (kcal/mol)		Comparable dangling end (kcal/mol)		Comparable dangling end (kcal/mol)	
5′PC<br>3′ G	-0.37	5′AC<br>3′ G	-0.9	5′GC<br>3′ G	-0.7
5′PA<br>3′ T	-0.63	5′AA<br>3′ T	-0.5	5′GA<br>3′ T	-0.6
5′ C<br>3′PG	0.07	5′ C<br>3′AG	-0.4	5′ C<br>3′GG	-0.4
5′ A<br>3′PT	0.09	5′ A<br>3′AT	-0.2	5′ A<br>3′GT	-0.2
5′ZC<br>3′ G	-0.64	5′CC<br>3′ G	-0.5	5′TC<br>3′ G	-0.6
5′ZA<br>3′ T	-0.80	5′CA<br>3′ T	-0.2	5′TA<br>3′ T	-0.3
5′ C<br>3′ZG	-0.11	5′ C<br>3′CG	-0.2	5′ C<br>3′TG	-0.8
5′ A<br>3′ZT	-0.17	5′ A<br>3′CT	-0.2	5′ A<br>3′TT	-0.2

Table S12. Stability of P-P or Z-Z terminal mismatches. The stabilities of analogous purine-purine or pyrimidine-pyrimidine mismatches are shown for comparison.

Terminal Mismatch	Stability (ΔG°_{37}; kcal/mol)	Analogous Mismatch	Stability (ΔG°_{37}; kcal/mol)	Analogous Mismatch	Stability (ΔG°_{37}; kcal/mol)
5′GP<br>3′CP	-0.44	5′GG<br>3′CG	-1.0	5′GA<br>3′CA	-1.0
5′TP<br>3′AP	-0.51	5′TG<br>3′AG	-0.4	5′TA<br>3′AA	-0.6
5′GZ<br>3′CZ	-0.76	5′GC<br>3′CC	-0.6	5′GT<br>3′CT	-0.9
5′TZ<br>3′AZ	-0.91	5′TC<br>3′AC	-0.2	5′TT<br>3′AT	-0.3

Table S13. Stability of mismatches on terminal Z-P pairs. The analogous stabilities for mismatches on C-G terminal pairs are shown for comparison.

Terminal Mismatch	Stability (ΔG°_{37}; kcal/mol)	Analogous Mismatch	Stability (ΔG°_{37}; kcal/mol)
5′GZ<br>3′GP	-0.52	5′GC<br>3′GG	-1.0
5′AZ<br>3′AP	-0.27	5′AC<br>3′AG	-1.0
5′CZ<br>3′CP	0.01	5′CC<br>3′CG	-0.6
5′GZ<br>3′AP	-0.23	5′GC<br>3′AG	-1.0
5′TZ<br>3′TP	-0.14	5′TC<br>3′TG	-0.9

Table S14. Single mismatch (1×1 internal loop) folding free energy changes.

Duplex	Loop Stability (kcal/mol)
5'GCCAPTTAA3'<br>3'CGGTCAATT5'	0.6
5'GCCAGTTAA3'<br>3'CGPTCAATT5'	-0.1
5'GCCAGTTAA3'<br>3'CPGTCAATT5'	-1.0
5'GCCAGTTAA3'<br>3'CGGTTAATT5'	0.7
5'GCTAGTTAA3'<br>3'CGGTCAATT5'	0.1
5'GTCAGTTAA3'<br>3'CGGTCAATT5'	-1.1
5'GCCAPTTAA3'<br>3'CGGTTAATT5'	1.6
5'GCTAGTTAA3'<br>3'CGPTCAATT5'	0.9
5'GTCAGTTAA3'<br>3'CPGTCAATT5'	-0.9
5'GZCAGTTAA3'<br>3'CAGTCAATT5'	-0.6
5'GCCAGTTAA3'<br>3'CAGTCAATT5'	-0.4

Table S15. Tandem mismatch (2×2 internal loop) folding free energy changes.

Motif	Motif Stability (kcal/mol)	Analogous Motif 1	Analogous Motif 1 Stability (kcal/mol)	Analogous Motif 2	Analogous Motif 2 Stability (kcal/mol)
5′TZZG3′<br>3′AZZC5′	-0.42	5′TCCG3′<br>3′ACCC5′	2.3	5′TTTG3′<br>3′ATTC5′	1.6
5′TPPG3′<br>3′APPC5′	0.58	5′TGGG3′<br>3′AGGC5′	1.6	5′TAAG3′<br>3′AAAC5′	1.5

Table S16. DNAzyme designs with or without P-Z pairs.

	Canonical DNA		DNA Including P-Z	
Design #	Time (s)	NED	Time (s)	NED
1	1573.3	0.064	1576.8	0.040
2	4634.0	0.074	1375.8	0.031
3	2858.7	0.071	1769.2	0.046
4	7342.7	0.041	1492.1	0.036
5	2313.9	0.104	1654.5	0.037
6	4067.5	0.138	1589.3	0.044
7	3378.9	0.078	1507.3	0.044
8	2049.3	0.100	1826.9	0.054
9	1461.8	0.203	1633.5	0.054
10	2636.4	0.174	1517.2	0.033
Mean:	3231.6	0.105	1594.2	0.042
Standard Deviation:	1770.2		134.1	
Best:		0.041		0.031

PZ Nearest Neighbor Parameter Tables:

The PZ parameter tables for use with RNAstructure are available upon request. The parameter tables expand upon the DNA $\Delta G°_{37}$ parameters found in RNAstructure. The tables provide parameter values, and the functional form is the same as that used by the Turner 2004 RNA $\Delta G°_{37}$ parameters as described in detail on the NNDB website: http://rna.urmc.rochester.edu .

Note that we determined parameters for $\Delta G°_{37}$. Because we did not determine parameters for $\Delta H°$, structure prediction must be performed at the default 37 °C.

There is a total of 20 files for the DNA+PZ parameters. All start with the prefix "PZ." This prefix is used by RNAstructure programs to identify the alphabet. Many of the command line programs provide the --alphabet flag, which can be used to specify the alphabet by adding "--alphabet PZ" to the command. In the file format, lines that start with "#" are comments. Other lines are interpreted by the software to read the parameters.

PZ.specification.dat contains the specification of the PZ alphabet. It contains nucleotides X, N, A, C, G, O, T, Z, and I. U can also be used, but it is interpreted as T. Lowercase nucleotides can be used, but these are not allowed to form base pairs. I is used to indicate a connection between two strands (a linker between two strands that are interacting). The allowed pairs are A-T, G-C, P-Z, and G-Z.

PZ.coaxial.dg contains the stabilities for flush coaxial stacking. This is the end-to-end stacking of two helix ends. These are modeled as the stability of a base pair stack (see PZ.stack.dg below).

PZ.coaxstack.dg contains the stabilities for coaxial stacks that are mediated by an intervening mismatch. These are end-to-end stacking of two helix ends, with a single mismatch intervening. This stack is across the mismatch and the adjacent helix, where the backbone is not continuous. This stability does not have any sequence dependence.

PZ.dangle.dg contains the stabilities for 5' and 3' dangling ends. These tables were expanded on the DNA table to include dangling ends on P-Z and G-Z pairs and to include dangling ends with P or Z. These extrapolations were informed by experiments reported in Table S8. The dangling end stabilities for these are reported in Tables S11A and S11B.

PZ.hexaloop.dg could contain the stabilities of hairpins of six unpaired nucleotides that are not well modeled. This table is empty for DNA and for DNA + PZ. It is needed for compatibility with RNA parameters, which include sequences in this table.

PZ.int11.dg contains stabilities for 1×1 internal loops, a.k.a. single mismatches. These were expanded from the DNA parameters to include P-Z and G-Z closing pairs and to include P or Z in the loop. These are sequence-dependent and are guided by experiments reported in Table S10 and analyses reported in Table S14. We use an experimental value, when available. When

the experimental value is not available, we map P-Z closure to entries for G-C closure and we map G-Z closure to entries for A-T. In the loops, we map Z to C and P to G. If the mapping results in a Watson-Crick-Franklin pair, we remap to an A-C mismatch, preserving the purine-pyrimidine orientation. We then lookup the entry as mapped to canonical nucleotides and further stabilize mismatches with P or Z by -0.6 kcal/mol, the mean additional stabilization observed for mismatches (Table S14) as compared to analogous AC mismatches.

PZ.int21.dg contains the stabilities of 2×1 internal loops, i.e. with 3 total unpaired nucleotides. Each sequence, including closing pairs, is represented. The table was expanded from the DNA parameters to include P and Z unpaired nucleotides and P-Z and G-Z closing base pairs. To extrapolate the stability of loops with P-Z and G-Z pairs, we map these to values with closing G-C and A-T pairs, respectively. Unpaired P and Z are mapped to loops with G and C, respectively. Loops with at least one P or Z unpaired nucleotides receive a stability bonus of -0.6 kcal/mol, which is informed by the single mismatch experiments reported in Table S10 and their analyses reported in Table S14.

PZ.int22.dg contains stability increments for 2×2 internal loops, i.e. internal loops with 2 unpaired nucleotides on each side of the loop. The table was expanded from DNA parameters to include P-Z and G-Z closing pairs and to include unpaired P or Z in the loop. These are extrapolated using experimental data reported in Table S10 and with analyses as reported in Table S15. When available, we used the experimental value in the table. When not available, we use the same remapping used for single mismatches (PZ.int11.dg) to look up an entry composed of canonical nucleotides. Then, for each mismatch with a P or Z, we stabilize by an additional -0.6 kcal/mol, which is the mean stabilization per mismatch observed for tandem mismatches as compared to analogous loops with canonical nucleotides (Table S15).

PZ.loop.dg contains loop closure free energy changes for internal, bulge, and hairpin loops. These tables are sequence independent and are identical to those used for DNA.

PZ.miscloop.dg contains sequence-independent terms used to calculate internal loop stabilities, hairpin loop, and multibranch loop stabilities. This table is unchanged from the DNA table.

PZ.stack.dg contains the helical stack parameters. This table was expanded from the DNA table to include P-Z and G-Z pairs and also to remove G-T pairs. This table uses the parameters in Tables 1 and 2. These table values were determined using the experimental data reported in Tables S3 and S5.

PZ.tloop.dg contains the stabilities for six hairpin loops with 4 unpaired nucleotides that are not well fit using the hairpin loop parameters. This table is unchanged from the DNA parameter set.

PZ.triloop.dg contains the stabilities for two hairpin loops with 3 unpaired nucleotides that are not well fit using the hairpin loop parameters. This table is unchanged from the DNA parameter set.

PZ.tstack.dg contains free energy increments for terminal mismatches in exterior loops. This table was expanded from the DNA parameters to include terminal P-Z and G-Z pairs and to include terminal mismatches with P or Z nucleotides. The parameters are guided by experimental results provided in Table S9, as analyzed and reported in Tables S12 and S13. The tables use the experimental value when available. For other mismatches, we extrapolated values using the following rules. P-P or Z-Z mismatches use the mean value for those measured, -0.5 or -0.8 kcal/mol, respectively. For all other terminal mismatches (other than P-P or Z-Z) on terminal P-Z, G-Z, or Z-G pairs or involving P or Z on A-T or G-C terminal pairs, we use the mean stability for mismatches on terminal Z-P pairs, -0.2 kcal/mol.

PZ.tstackcoax.dg contains sequence-dependent terminal mismatch parameters for mismatches stacking in a mismatch-mediated coaxial stack. These parameters are equal to the PZ.tstack.dg values.

PZ.tstackh.dg contains stability increments for the first mismatch in a hairpin loop. This table was expanded from the DNA parameters to include P-Z and G-Z closing pairs and to include P or Z in the first mismatch. This table is identical to PZ.tstack.dg.

PZ.tstacki.dg contains stability increments applied to first mismatches in internal loops larger than 2×2 internal loops (other than 2×3 internal loops). These were expanded from the DNA parameters by including P-Z and G-Z closing pairs and by including mismatches with P or Z. To extrapolate the new parameters, we used the values in PZ.tstack.dg.

PZ.tstacki1n.dg contains stability increments for first mismatches in 1×n internal loops, i.e. loops with 1 unpaired nucleotide on one side and n (n>2) on the other side of the loop. The table was expanded from the DNA table to include P-Z and G-Z closing pairs and to include P or Z in the first mismatch. This table uses the same 0 kcal/mol increment for all first mismatches as the DNA table.

PZ.tstacki23.dg contains stability increments applied to first mismatches in 2×3 internal loops. These were expanded from the DNA parameters by including P-Z and G-Z closing pairs and by including mismatches with P or Z. To extrapolate the new parameters, we used the values in PZ.tstack.dg.

PZ.tstackm.dg contains stability increments for the terminal mismatches in multibranch loops. This table was expanded from the DNA parameters to include P-Z and G-Z pairs and to include P or Z in the mismatch. This table is identical to PZ.tstack.dg.
